# The Scatter Search Based Algorithm for Beam Angle Optimization in Intensity-Modulated Radiation Therapy

**DOI:** 10.1155/2018/4571801

**Published:** 2018-06-03

**Authors:** Ali Ghanbarzadeh, Majid Pouladian, Ali Shabestani Monfared, Seied Rabi Mahdavi

**Affiliations:** ^1^Department of Medical Radiation Engineering, Tehran Science and Research Branch, Islamic Azad University, Tehran, Iran; ^2^Department of Biomedical Engineering, Tehran Science and Research Branch, Islamic Azad University, Tehran, Iran; ^3^Cancer Research Center, Medical Physics Department, Babol University of Medical Sciences, Babol, Iran; ^4^Radiobiology Research Center, Department of Medical Physics, Iran University of Medical Sciences, Tehran, Iran

## Abstract

This article introduces a new framework for beam angle optimization (BAO) in intensity-modulated radiation therapy (IMRT) using the Scatter Search Based Algorithm. The potential benefits of plans employing the coplanar optimized beam sets are also examined. In the proposed beam angle selection algorithm, the problem is solved in two steps. Initially, the gantry angles are selected using the Scatter Search Based Algorithm, which is a global optimization method. Then, for each beam configuration, the intensity profile is calculated by the conjugate gradient method to score each beam angle set chosen. A simulated phantom case with obvious optimal beam angles was used to benchmark the validity of the presented algorithm. Two clinical cases (TG-119 phantom and prostate cases) were examined to prepare a dose volume histogram (DVH) and determine the dose distribution to evaluate efficiency of the algorithm. A clinical plan with the optimized beam configuration was compared with an equiangular plan to determine the efficiency of the proposed algorithm. The BAO plans yielded significant improvements in the DVHs and dose distributions compared to the equispaced coplanar beams for each case. The proposed algorithm showed its potential to effectively select the beam direction for IMRT inverse planning at different tumor sites.

## 1. Introduction

Intensity-modulated radiation therapy (IMRT) is an advanced form of the state-of-the-art three-dimensional conformal radiation treatment that improves therapeutic ratios. In IMRT, the radiation beam is modulated by a multileaf collimator. Intensity-modulated beams from different directions are irradiated to achieve a higher degree of uniform dosage for the planning target volume (PTV) and to decrease the dose as much as possible to the organs at risk (OAR) [1]. Conventionally, IMRT treatment planning starts with beam angle selection and is followed by determination of the intensity profiles for preselected beams using an inverse planning method [2, 3].

Currently, in many locations, beam angle selection for IMRT treatment planning is done simply by choosing equiangular spaced beams or through time-consuming trial and error based on the experience of the treatment planners. These methods provide little chance for arriving at the optimal beam configuration because the total dose distribution is affected by the complexity of the profile intensities from every beam direction [4–6].

Selection of the optimal beam direction significantly improves the quality of IMRT treatment both for tumor coverage and in OAR sparing [4, 5, 7–11]. Briefly, determining the optimum beam configuration is a combinatorial optimization problem, in which the best angle configuration is obtained from the results of subproblem solving such as fluence map optimization (FMO) [12]. FMO optimizes the intensity profile for each selected beam angle to ensure that the resulting treatment plan meets the prescribed dose distribution and clinical criteria [13–15].

Beam angle optimization (BAO) is a computationally intensive problem for a number of reasons. First, the search space of the solutions is huge, requiring enumeration of all possible beam orientation combinations. For example, when choosing 4 angles out of 36 candidate beam angles, *C*_4_^36^ = 58905 possible combinations exist. Second, any change in a beam configuration requires recalculation and reoptimization of intensity maps, itself a time-consuming process. Third, many local minima (maxima) will appear in the objective function [15–18].

The complexity of the beam angle problem has prompted a wide body of research on automatization of the process in two past decades. There are two important methodologies for solving the beam selection problem. The first is the scoring method, where scores are assigned to beam angles based on the different beam angle ranking functions, the beam-eye view [19], geometric algorithms [20], and dosimetric information [21, 22]. When beams with higher scores are selected, the intensity of each beamlet can be obtained by FMO. This kind of algorithm is very efficient computationally because the interdependence of the multiple modulated beams is neglected during beam angle selection. There is no guarantee, however, that the beam set is optimal because beam selection in this method is not based on the optimal response of FMO and the interplay between the beam sets [4, 19].

The second method is beam configuration based on the objective function value of the FMO which measures plan quality. This framework is very time-consuming because the FMO problem must be solved for each beam configuration to obtain the optimal objective function value. If the function becomes trapped in the multiple local minima of the problem, it may lead to a suboptimal solution [17, 18]. Metaheuristic and stochastic algorithms have been used to escape from the local minimum to obtain a global optimum and the problem can be solved efficiently using simulated annealing [4–7, 23], genetic [23–26], particle swarm optimization [27], pattern search [28], and branch and prune algorithms [29].

Due to the multiple local optimal solutions and nonconvex nature of the BAO, the current study chose the Scatter Search Based Algorithm as the optimization technique as a rapid method to reach the global optimum. The Scatter Search (ScS) method is an optimization derivative-free algorithm based on the sparse grid numerical integration. This algorithm is suitable for a pure and mixed integer nonlinear objective function for which calculation of the gradient is impossible and evaluation of its value is time-consuming [30].

The present study was undertaken to investigate beam angle selection by a new framework. This is the first time that the Scatter Search Algorithm has been incorporated with FMO to search along a discrete-angle candidate pool to find the optimal angle set. The performance of the selected beam angle selection framework was verified using a simulated box phantom with obvious optimal beam angles. The plan quality was compared with a typical equispaced beam selection treatment plan for a TG-119 phantom and a prostate case.

## 2. Materials and Methods

**Figure 1:**
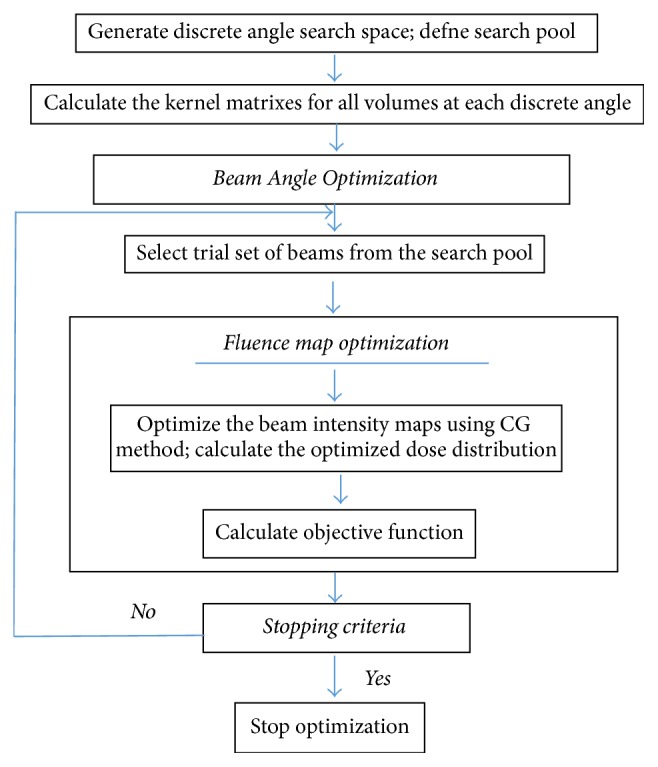
Flowchart of beam angle selection algorithm.

The goal was to find a set of beam directions and corresponding beamlet intensities that could produce the desired conformal dose distribution. The proposed beam angle selection algorithms use two optimization loops. First, for beam orientation selection, the BAO algorithm suggests a small set of beam orientations denoted by *θ* among a beam candidate pool denoted as Θ. Second, the beam intensity map is fluence-optimized to determine the corresponding dose distribution inside the body volume. This dose distribution is used to evaluate the performance of the trial solution using the value of the objective function (*F*(*θ*)). The beam intensity maps are first optimized. Details of the algorithm are provided in a flowchart in Figure 1.

### 2.1. BAO Problem Formulation

The angle search space in this study covered an entire 360° coplanar gantry angle that is divided into equally spaced directions. It is set to 10° for the simulated case and 5° for other cases and the collimator angle and coach angle are kept fixed. The combinations of these discrete angles are referred to as trial angles [25].

Let Θ be a set of candidate angles that contain the combination of *n* coplanar beams defined as Θ = (*θ*_1_, *θ*_2_,…, *θ*_*N*_) in which *N* is the total number of feasible beam orientations. Each beam configuration (*θ*_*i*_) is made up of *n* coplanar beam directions *θ* = (*b*_1_, *b*_2_,…, *b*_*n*_) and each beam angle *b*_*i*_ is divided into 1 × 1 cm^2^ beamlets on the isocenter plane along the irradiation. The beamlet intensities of angle *b*_*i*_ are ${\vec{x}}_{i}=\left(x_{1},x_{2},\ldots ,x_{k}\right)$. In FMO, the weights (intensities) of the rays are optimized. Once the optimized intensity maps are determined, the corresponding objective function of the current beam configuration can be calculated. The BAO problem can be stated mathematically as

(1) min Fθisubject  to θi∈Θ.

Objective function *F*(*θ*_*i*_) is the optimal FMO value resulting from the angle set specified by (*b*_1_, *b*_2_,…, *b*_*n*_) in the FMO problem and is formulated such that the lower objective function values correspond to improved solutions. During optimization, the algorithm provides candidate starting points for any gradient-based local solver. This process is called Scatter Search. The gradient-based local solver seeks the answer with the best fitness value near the sparse starting points [31].

### 2.2. FMO Problem Formulation

In the FMO formulation, each beam angle can be treated as hundreds of smaller beamlets, each of which having its own radiation intensity (called a fluence). The modulation of these fluences for the beamlets in a set of beams allows for precise control of radiation delivery to the patient. To accelerate each iteration of dose calculation, a strategy similar to that reported by Djajaputra et al. [6] was used, in which the dose deposited in voxel *i* by an IMRT beam is given by *D*_*i*_ = ∑*K*_*im*_*x*_*m*_, where *x*_*m*_ is the weight for the *m*th beamlet. Kernel *K*_*im*_ is the “dose kernel” or “dose matrix” deposited by each beamlet *j* at unit intensity *K*_*ij*_ for each voxel *i* in structure* s*.

Several types of objective functions exist and are implemented in clinical IMRT optimization problems. A quadratic objective function of the difference between the actual and desired dose as introduced by Oelfke and Bortfeld [32] was used to find the ideal fluence modulation $\vec{x}=(x_{1},x_{2},\ldots ,x_{n})$ for a given *N*_ray_ beam ensemble *θ*_*i*_. The parameter notations are shown in Notations.

The FMO problem for a given set of beams *θ* is as follows:

(2) min Fx→=∑i=1NTPTVpiDix→−Dipres2+∑j=1NOAR∑i=1NTjpiDix→−Dimax+2

(3) subject  to Dix→=∑m=1NrayKim·x→m,

(4)  x→m≥0.

The positive operator ensures that only violated constraints contribute to the objective function; that is, ⌊*x*⌋_+_ = *x* for *x* > 0 and ⌊*x*⌋_+_ = 0; otherwise, negative weights of beamlets will not be acceptable in the optimization. A hard constraint was thus defined, which will not violate (4). The final objective value shows a difference between the desired and calculated dose distribution which denotes the quality of the beam angle sets.

Optimization aims to minimize the dose difference between the prescribed and calculated dose distributions. A conjugate gradient (CG) algorithm is used to solve the FMO problem in the proposed framework. CGs are beneficial from the computational standpoints. The problem may be trapped in local minima, because CG is a local search method [27, 33], but several investigators have demonstrated that those minima are very close to one another and the resulting treatment plans are almost the same [17, 18, 33].

### 2.3. The Scatter Search Based Method

The investigation algorithm attempts to find the global solution by starting a local solver from multiple start points in search space. The algorithm uses multiple start points to sample multiple basins of attraction [31, 34].

The Scatter Search Based Algorithm performs the following steps:

1. The Scatter Search based Algorithm runs a local solver (in MATLAB, fmincon is this local solver) from the start point which was given the problem structure. If this run converges, algorithm records the start point and the end point for an initial estimate on the radius of a basin of attraction.
2. Generate trial points.
3. The proposed algorithm uses the Scatter Search Algorithm to generate a set of trial points that are potential start points.
4. Scatter Search (ScS) is a population-based metaheuristic algorithm that operates on a set of solutions called the reference set or population. Reference set is generated from a population of solution. Then, in the improvement procedure, the solutions in this reference set are combined to get starting solutions, whose result may update the reference set and even the population of solutions from iteration to iteration. ScS is an evolutionary algorithm (EA) because it builds, maintains, and evolves a set of solutions throughout the search. In contrast to other evolutionary methods like genetic algorithms (GA), in Scatter Search the selection of the parents is made using a deterministic method called Subset Generation Method but, in GA, parents are chosen following a random sampling scheme [35, 36]. Implementation of Scatter Search is based on the following steps:
  o Generate a starting set of solution vectors by heuristic processes designed for the problem considered and designate a subset of the best vectors to be reference solutions.
  o The trial solution improves to transform into enhanced trial solution.
  o The reference set updates based on the best of solutions found. Solutions are ranked according to their quality or their diversity.
  o Linear combinations of subsets of the current reference solutions generate a new combined solution and new reference set.
  o A collection of the best solutions are starting points for new heuristic processes of step (I). Repeat these steps until reaching a specified iteration limit [36, 37].
5. The algorithm evaluates the score function of a set of trial points. It then takes the point with the best score and runs local solver from that point. The algorithm removes the set of trial points from its list of points for examination.
6. Initialize basins and counters: the algorithm heuristic assumption is that basins of attraction are spherical. The initial estimates of basins of attraction for the solution point from *x*0 and the solution point from Stage 1 are spheres centered at the solution points. The radius of each sphere is the distance from the initial point to the solution point. These estimated basins can overlap.
7. There are two sets of counters associated with the algorithm. Each counter is the number of consecutive trial points that
  - lie within a basin of attraction, where there is one counter for each basin,
  - have score function greater than localSolverThreshold. For a definition of the score. All counters are initially 0.
8. Begin main loop.

The Scatter Search Based Algorithm repeatedly examines a remaining trial point from the list and performs the following steps. It continually monitors the time and stops the search if elapsed time exceeds MaxTime seconds.

After reaching MaxTime seconds or running out of trial points, the algorithm creates a vector of Global Optimal Solution objects and orders the elements of the vector by objective function value, from lowest (best) to highest (worst) [34].

## 3. Results

**Figure 2:**
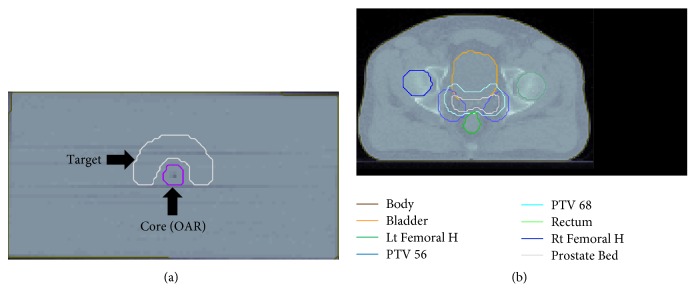
Axial view CT and structures for both cases using CORT dataset: (a) TG-119 phantom; (b) prostate.

The proposed beam angle selection algorithm was tested using simulated and clinical cases. These involved a box phantom to benchmark the framework for finding the best optimal solution along with a TG-119 phantom and a prostate case to compare the plan quality of the optimal angles with equispaced beam angle selection (Figure 2). Beam angle selection algorithms were coded in Matlab R2016R and run on a laptop with Intel Core i7 CPU-6700HQ @ 2.6 GHz with 16 GB of main memory.

Dose volume histogram (DVH) analysis was used to evaluate the quality of proposed treatment plans. Some plan indices that are routinely used to describe a plan are as follows: Dose homogeneity index (HI): analyze the uniformity of dose distribution within the target volume as [38]

(5) $$\begin{array}{c} HI=\frac{D_{5}-D_{95}}{D_{p}}\times 100, \end{array}$$

where *D*_5_ and *D*_95_ are the minimum doses at 5% and 95% of the target volumes, respectively. They denote the maximum and minimum dose of the target, respectively, with *D*_*p*_ denoting the prescribed dose. The ideal value for HI is zero when *D*_5_ and *D*_95_ are equal [39]. The conformity index (CI) was defined by Van't Riet et al. (1997) as [40]

(6) $$\begin{array}{c} CI={CI}_{1}\cdot {CI}_{2}=\frac{V_{t,ref}}{V_{t}}\cdot \frac{V_{t,ref}}{V_{ref}}, \end{array}$$

where *V*_*t*_ denotes the target volume, *V*_*t*,ref_ denotes the target volume covered by the reference isodose, and *V*_ref_ denotes the volume covered by the reference isodose. CI ranges from 0 to 1 with the ideal being CI = 1.

CI_1_ expresses the fraction of the target volume that receives at least 95% of the prescribed dose. This term is equal to or slightly lower than one for ideal plans. CI_2_ indicates how high a dose (greater than 95% of the prescribed dose) is delivered adjacent to the target volume. A lower value means that the higher dose has spilled around the tumor volume. This term was considerably lower for the optimum plan.

### 3.1. Simulated Case

The simulated box phantom with defined optimal angles was used to benchmark the BAO algorithm to find the optimal angle set. The simulated case contains a cubic PTV with four obvious optimal beam angles (0°, 90°, 180°, and 270°) [25]. The parameters and the shape of the simulated case are based on the CORT dataset (common optimization for radiation therapy) [41].

The voxel size was set to 0.3 × 0.3 × 0.5 cm^3^. The beamlet size was set to 1 × 1 cm^2^ on the isocenter plane. The objective function parameters were selected as follows: PTV prescribed dose = 30 Gy, penalty factor of PTV = 1000, body maximum dose = 20 Gy, and body penalty factor = 100. The BAO algorithm was run to find four coplanar 6-MV photon beams.

**Figure 3:**
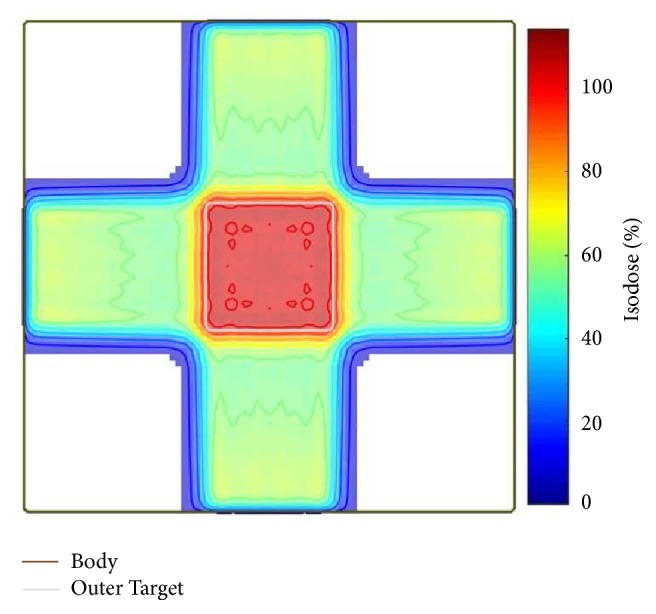
Box phantom isodose from optimal angles of 0°, 90°, 180°, and 270°.

To the best of our knowledge, the optimal angles of box phantom are equispaced. The starting point of the algorithm and upper and lower bounds of each angle did not include the 0°, 90°, 180°, and 270° beam angles to allow full testing of algorithm performance. The proposed BAO algorithm found the expected optimal angles (i.e., 0°, 90°, 180°, and 270°). The dose distribution of the optimal angles is shown in Figure 3.

### 3.2. TG-119 Case

**Figure 4:**
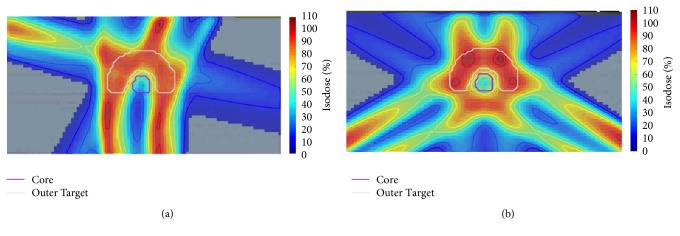
TG-119 phantom case. Comparison of axial dose distribution obtained in (a) optimal angle set plan (30°, 180°, and 285°) and (b) equiangular plan (0°, 120°, and 240°).

The TG-119 phantom with a concave PTV and cylindrical OAR is shown in Figure 2. The simulated TG-119 phantom in the CORT dataset was used. The voxel size was set to 0.3 × 0.3 × 0.25 cm^3^. The numbers of target voxels and total patient voxels were equal to 7,429 and 599,440, respectively. The objective function parameters were set as follows: prescribed dose to PTV = 50 Gy with a penalty factor of 1000, OAR max dose = 30 Gy with a penalty factor of 300, and body max dose = 30 Gy with a penalty factor of 100. For this case, the BAO algorithm considered three optimal beam angles (30°, 180°, and 285°) from the coplanar candidate orientations. To show the effectiveness of the algorithm in reaching a better beam orientation, the dose distribution of the optimal plan was compared with equispaced beams as illustrated in Figure 4.

The reason for the fact that comparison of the performance of the algorithm was evaluated with the equispaced beams is because such beams are commonly used in clinical treatment planning and are clinically acceptable [10]. The planning target coverage and OAR sparing of the plans were evaluated with DVH and some of the clinical matrices (e.g., homogeneity and conformity indices).

**Figure 5:**
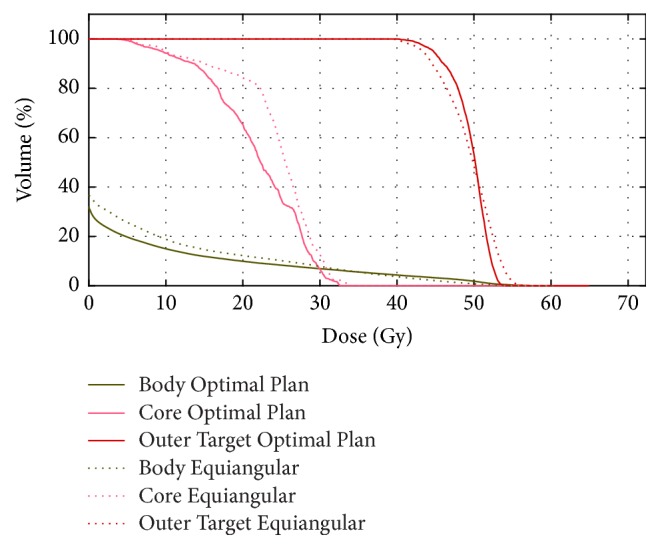
DVH comparison for TG-119 phantom case of equiangular and optimal beam angle set plans. Three coplanar 6 MV photon beams were used for both plans.

The DVHs (Figure 5) showed that the OAR received a lower dose in the optimized plan than in the manual plan; furthermore, the uniformity of the dose in PTV was slightly improved in the optimal plan.  Table 1 shows that the BAO algorithm produced better-quality treatment plans in terms of target coverage (CI), target dose homogeneity (HI), and OAR sparing (mean and maximum dose of core).

### 3.3. A Clinical Case: Prostate Tumor

For the prostate case, the CORT dataset was used. In this case, two PTVs with prescribed doses of 56 and 68 Gy were defined, which were surrounded by the OARs of the rectum and bladder (Figure 2). The voxel size was set to 0.3 × 0.3 × 0.3 cm^3^ and the voxel numbers of the target and patient body in the image were 9491 and 690,373, respectively. The optimization objective function parameters are shown in Table 2.

**Figure 6:**
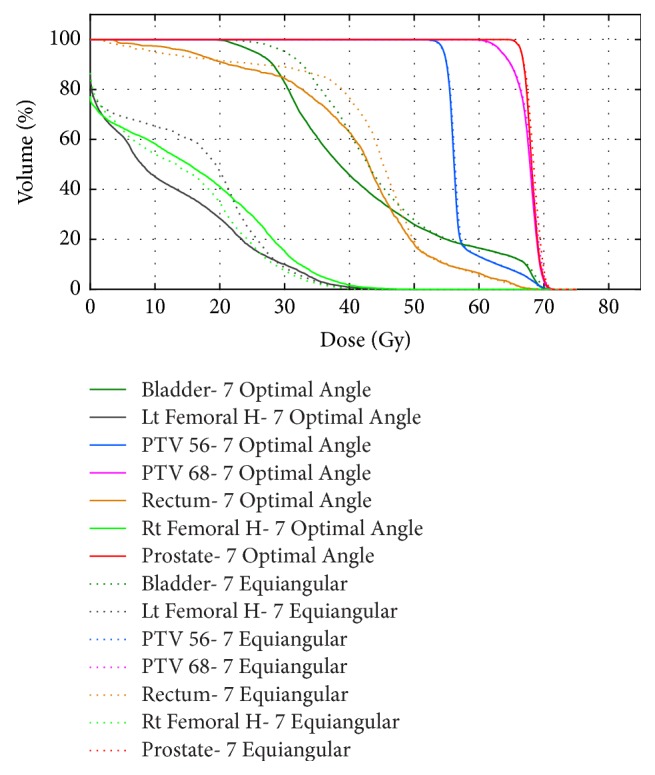
Comparison of DVHs of seven-beam angle selection plan and BAO algorithm plus seven-beam equiangular plan.

**Figure 7:**
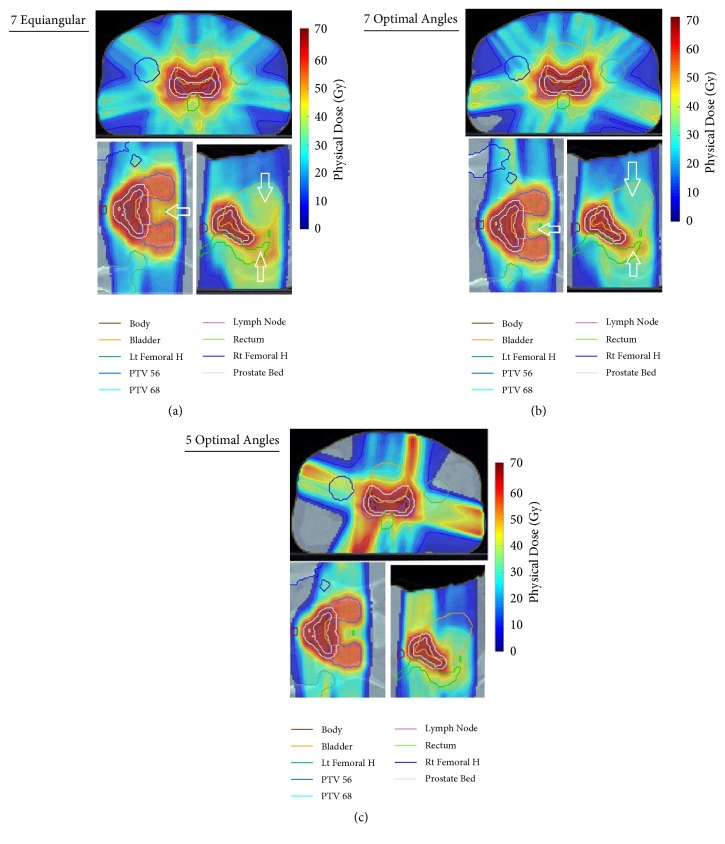
Dose distributions for prostate case in axial, coronal, and sagittal views: (a) seven-angle equiangular plan (0°, 50°, 100°, 150°, 200°, 255°, and 305°); (b) seven-angle optimal plan (5°, 50°, 110°, 200°, 225°, 260°, and 320°); (c) five-angle optimal plan (5°, 105°, 185°, 210°, and 290°).

The seven angles selected by the BAO algorithm were 5°, 50°, 110°, 200°, 225°, 260°, and 320°. It was observed through several runs of the problem that some beam angles in the configuration changed slightly, but the overall objective function value did not change significantly (Figure 6) compared to the DVHs of the sets of seven optimal coplanar beams and seven equispaced beams. The target dose did not change significantly, while the bladder and rectum doses decreased. These plans were optimized using the same dose prescription for the objective function.  Figure 7 shows the dose distributions and confirms that the BAO improved the quality of the plan in comparison with the equispaced method. Quality indicator values for both the optimal beam angle treatment plan and the reference plan (equispaced beam selection) are listed in Table 3.

**Figure 8:**
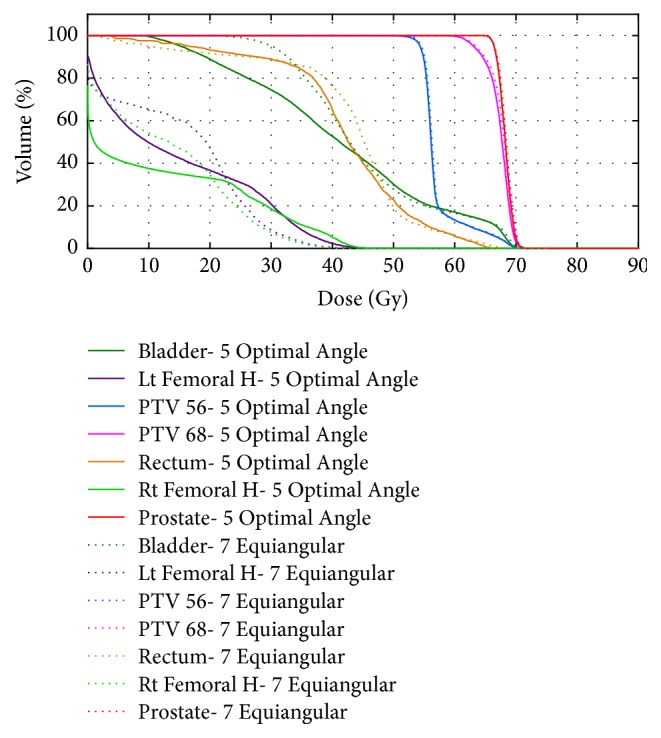
DVHs for the prostate case. Solid lines denote the five-beam plan generated by BAO algorithm. Dotted lines denote the results of the treatment plan with seven equiangular beams.

Comparison of the DVH of the five optimal angles and seven equiangular plans shows that target coverages were similar, but the BAO plans delivered a smaller dose to the bladder and rectum (Figure 8).  Figure 7 shows the plan using five beams to achieve the best possible dose distribution with quality as good as or better than a plan with a larger number of equispaced beam angles.

## 4. Discussions

This paper introduces a Scatter Search Based Algorithm to solve the problem of beam angle selection in IMRT planning. The results of testing on a simulated box phantom showed the ability of the proposed algorithm to reach an optimal coplanar beam configuration, which is the benchmarking framework for receiving optimal beam angles for IMRT treatment planning. As in previously published works in BAO, the proposed framework can improve the quality of plan by choosing the preferable beam orientation, but there is no way to determine whether or not the solutions of BAO are a global optimum or perhaps suboptimal.

The ability of proposed beam angle selection framework to clinically improve complicated IMRT plans (TG-119 phantom and prostate cases) was compared to manual equispaced beams. The DVH, dose distribution, and quality indices of the plans confirmed that the optimum beam angle set improved OAR sparing while guaranteeing target coverage and dose uniformity.  Table 1 showed a decrease of approximately 2.52 and 1.32 Gy for mean and maximum core doses (OAR) in the TG-119 case, respectively. Table 3 shows that the mean doses for the bladder and rectum of the optimal plan decreased by 3.22 and 2.03 Gy, respectively, over those of the equiangular plan.

The stochastic and heuristic proposed Scatter Search Based Algorithm for solving the beam angle optimization problem was suitable due to the nonconvex nature of the problem with multiple local minima [15, 16]. The proposed algorithm analyzes feasible solutions by running multiple starting points selected from the scattered base by a search poll. This allows the algorithm to overcome local minima.

The computation time of the proposed framework increased with control of the main factors that influence beam selection time such as the BAO coupled with FMO, the size of the initial candidate beam configuration, number of targets and OAR voxels, and size of the dose matrix. The algorithm spent the most time finding an intensity map of each beam configuration because the objective function for beam angle optimization was based on the optimal dose distribution obtained from each beam configuration. FMO was used to conjugate the gradient algorithm, which is preferred to other algorithms in many studies because of its faster convergence [33]. The CG, however, is a local search algorithm that can be trapped in the local minima and make suboptimal plans. The dose distribution obtained from the optimal intensity of solving FMO must be calculated by scoring each beam angle set during BAO because the fast gradient algorithm can significantly decrease computing time.

Choosing an optimum number of beams improves the quality of the IMRT treatment plan. In the proposed framework, the number of beams was selected before BAO based on the complexity level of the given case [4, 9]. Moreover, noncoplanar beam angles were not investigated in this study, which may further improve the quality of the plan.

The beam angle discretization resolution was set at 10° for the box phantom case and 5° for the TG-119 and prostate case. Many investigators have discussed the influence of this resolution on the final solution for the BAO algorithm and computation time [6, 42]. To speed up the algorithm, the search space size can be reduced by prior knowledge, such as selection of more beams in favorable directions and vice versa. An alternative method to reduce the feasible beam direction is to discrete the coplanar pool using a reasonable space of, for example, 10 instead of 5, to allow half candidate directions that significantly decrease the number of possible beam configurations.

Figures 7 and 8 showed that a lower number of optimal angles in the plan can reach dose distributions that are as good as plans using a greater number of equispaced orientations. Furthermore, a plan with a small number is more highly desirable from the clinical perspective to limit the volume of normal tissue being irradiated or simplifying treatment delivery to shorten treatment time and hence lower potential error caused by patient movement during dose delivery [4, 5, 11].

## 5. Conclusions

The proposed angle selection algorithm is able to provide better beam orientation configurations for IMRT, which will spare OARs and achieve better target volume coverage. The small number of optimized orientations can obtain results similar to plans with a greater number of manual beam directions, which means easier quality assurance and a decrease in treatment time and patient setup error. The main advantage of this method is that the Scatter Search Algorithm fits with the nonconvex nature of the problem and can search all space in a short time to choose the beam angles, which may be very helpful for routine clinical usage. Also, the proposed algorithm can run any IMRT case and can fulfill different clinical desires by changing the parameters of the objective function.

## Figures and Tables

**Table 1:** Comparison of plan quality indices for selection of three angles in TG-119 phantom case.

VOI	*Optimal plan*	*Equiangular plan*
Target		
*D*_5_ [Gy]	52.74	54.19
*D*_95_ [Gy]	44.73	43.3
HI (%)	16.02	21.77
CI	0.478	0.321
Core (OAR)		
Mean dose [Gy]	21.82	24.34
Max. dose [Gy]	32.63	33.95

**Table 2:** Objective function parameters for all structures considered for BAO of the prostate case.

VOI	VOI type	Objective function	Penalty	Prescribed dose [Gy]	Mean dose [Gy]	Maximum dose [Gy]
PTV 56	Target	Square deviation	1000	56	-* *-* *-	-* *-* *-
PTV 68	Target	Square deviation	1000	68	-* *-* *-	-* *-* *-
Bladder	VOI	Square overdosing	300	-* *-* *-	45	-* *-* *-
Rectum	VOI	Square overdosing	300	-* *-* *-* *-	45	-* *-* *-
Body	VOI	Square overdosing	100	-* *-* *-	-* *-* *-	70

**Table 3:** Comparison of plan quality indices for seven-angle selection of prostate case.

VOI	*Optimal plan*	*Equiangular plan*
PTV 56 (Target)		
*D*_5_ [Gy]	67.35	67.33
*D*_95_ [Gy]	54.56	54.55
HI (%)	22.83	22.82
CI	0.4153	0.3548
PTV 68 (target)		
*D*_5_ [Gy]	69.8	70.06
*D*_95_ [Gy]	63.45	63.44
HI (%)	9.3359	9.7482
CI	0.8243	0.7647
Bladder (OAR)		
Mean dose [Gy]	42.32	45.54
Max. dose [Gy]	71.19	71.45
*D*_5_ [Gy]	68.33	68.78
*D*_95_ [Gy]	25.39	30.13
Rectum (OAR)		
Mean dose [Gy]	40.92	42.95
Max. dose [Gy]	69.37	69.94
*D*_5_ [Gy]	61.36	60.94
*D*_95_ [Gy]	15.25	9.89

## Notations

### Notations and Definitions of Parameters Considered for the Objective Function

$\vec{x_{m}}$: Intensity of *m*th ray
*p*_*i*_: Penalty coefficient in voxel *i*
$D_{i}\left(\vec{x}\right)$: The calculated dose of the *i*th point in the volume
*D*_*i*_^pres^: The prescribed dose in the planning target volume
*D*_*i*_^max^: The tolerance dose in organs at risk (OARs)
*N*_OAR_: The total number of the OARs
NT_*j*_: Number of voxels in *j*th OARs
NT_PTV_: Number of voxels in the target
*N*_ray_: The total number of the rays
*K*_*im*_: The dose deposited to the *i*th point (voxel) from the *m*th ray with a unit beamlet weight.
